# The Need for Continued Development of Ricin Countermeasures

**DOI:** 10.1155/2012/149737

**Published:** 2012-03-26

**Authors:** Ronald B. Reisler, Leonard A. Smith

**Affiliations:** ^1^Division of Clinical Medicine, Clinical Research Management Inc., Team Ke'aki Tech, United States Army Medical Research Institute of Infectious Diseases, 1425 Porter Street, Fort Detrick, MD 21702-5011, USA; ^2^Division of Toxinology, United States Army Medical Research Institute of Infectious Diseases, 1425 Porter Street, Fort Detrick, MD 21702-5011, USA

## Abstract

Ricin toxin, an extremely potent and heat-stable toxin produced from the bean of the ubiquitous *Ricinus communis* (castor bean plant), has been categorized by the US Centers for Disease Control and Prevention (CDC) as a category B biothreat agent that is moderately easy to disseminate. Ricin has the potential to be used as an agent of biological warfare and bioterrorism. Therefore, there is a critical need for continued development of ricin countermeasures. A safe and effective prophylactic vaccine against ricin that was FDA approved for “at risk” individuals would be an important first step in assuring the availability of medical countermeasures against ricin.

## 1. Introduction

In the aftermath of September 11, 2001, it has become increasingly clear that there is a need to enhance readiness against attack from both state sponsors and nonstate sponsors of bioterrorism. Ricin toxin, an extremely potent and heat-stable toxin produced from the bean of the *Ricinus communis* (castor bean plant) [1], has been categorized by the US Centers for Disease Control and Prevention (CDC) as a category B biothreat agent for biological warfare and bioterrorism [2]. In fact, according to Cookson and Nottingham, ricin was code named compound W and considered for weaponization during the US offensive Biological Warfare Program [3]. The US intelligence community believes that ricin was a component of the biowarfare program of the former Soviet Union, Iraq, and possibly other countries as well [4, 5].

Ricin toxin is relatively easy to produce and potentially lethal when delivered orally, intramuscularly, or through inhalation [4]. While the primary large-scale threat to US military personnel would be through powdered material that could be inhaled, ricin has been used successfully to assassinate individuals, to carry out suicide, and in 2003-2004, to terrorize US postal and Senate workers [4]. This paper reviews the rationale for development of ricin countermeasures and the progress toward achieving effective ricin countermeasures.

## 2. Background

Ricin is a 65 kilodalton (kDa) polypeptide toxin comprised of two dissimilar polypeptide chains (an A-chain and a B-chain) held together by a disulfide bond [1, 4, 5]. The A-chain, ~32 kDa, targets the ribosome and is therefore a potent inhibitor of protein synthesis [4, 5]. Consequently, the A-chain has been classified as a ribosome-inactivating protein (RIP) [4, 5]. The B-chain, ~34 kDa, is a galactose or an N-acetylgalactosamine-binding lectin that attaches to cell-surface receptors [4, 5]. After binding and subsequent endocytosis, the holotoxin travels through the Golgi apparatus to the endoplasmic reticulum where the disulfide bond linking the A and B chains is reduced. Once the disulfide bond is broken, the A-chain molecule is transported to the cytosol where it inactivates the ribosome. In fact, just one ricin molecule per cell may be sufficient to permanently inhibit that cell from performing essential cellular protein synthesis [6].

Ricin holotoxin is lethal in mice, rabbits, and monkeys at parenteral doses of 5–25 *μ*g/kg [4]. By inhalation, ricin has an LD_50_ in mice, rabbits, and monkeys of 3–17 *μ*g/kg, and by ingestion it has an LD_50_ of 20 mg/kg [4]. When ricin toxin A (RTA) chain is separated from ricin toxin B (RTB) chain and is administered parenterally to mice, it has limited toxicity at lower doses. RTA is approximately 1000-fold less toxic than natural ricin at lower doses when administered parentally to mice [7].

Ricin toxin is a potential threat to humans by three distinct routes: aerosolized ricin via the pulmonary system, food and water via the gastrointestinal system, and bioweaponized munitions including improvised explosive devices, via skin wounds [8, 9]. For more than 120 years, researchers have been working on ways to both develop prophylaxis against ricin exposure and to effectively treat ricin postexposure [4].

## 3. Early Work on Ricin Vaccine and Pretreatment for Ricin Exposure

Initially, as early as the 1890s, Paul Ehrlich vaccinated mice with oral doses of ricin and then subsequently challenged the mice with subcutaneous lethal doses of ricin [10]. Later, in the 1940s, a formalin-inactivated holotoxin vaccine was developed by the US Army that enhanced survival in animals [11]. This vaccine candidate did not progress past preclinical testing. Pretreating animals with passive transfer of either IgG polyclonal antibody [12–14] or monoclonal directed against RTA, appeared to effectively protect them from lethal parenteral challenge to ricin [15–17]. Protection against a lethal dose of aerosolized ricin with passive transfer of either IgG polyclonal or monoclonal antibody directed against RTA has proved to be more difficult to achieve.

## 4. Progress toward a Prophylactic Ricin Vaccine

### 4.1. US Army Ricin Vaccine Development (1990s)

Past attempts to produce a ricin vaccine with an Alhydrogel-adsorbed ricin toxoid [18–20] and a deglycosylated RTA (dgRTA) vaccine (Lot 01-0419964, PerImmune) [20–22] suggested that although both products can induce protective immunity against the toxin in animals, their use as vaccines was limited by safety concerns raised during preclinical development, the tendency to self-aggregate and precipitate from solution, and difficulties associated with process and product characterization during manufacturing. Thus, these vaccine candidates were limited to pre-clinical testing and never progressed to human clinical trials.

### 4.2. RiVax Recombinant Vaccine

RiVax, an investigational recombinant protein RTA vaccine, was developed based on studies with ricin and RTA [23, 24]. RiVax is essentially RTA with two simple amino-acid substitutions, one in the LDV amino acid sequence {amino acid residues 74–76}, hypothesized to play a key role in intact RTA-induced Vascular Leak Syndrome (amino acid 76: valine replaced by methionine), and the other in a ribotoxic site (amino acid 80: tyrosine replaced with alanine) [23]. RiVax was found to have sufficient preclinical safety data to proceed to a human phase I dose-escalating study [24, 25]. The human phase I study was designed as follows: 15 healthy volunteers (three groups of five) were vaccinated three times with intramuscular (IM) RiVax (doses were either 10 *μ*g, 33 *μ*g, or 100 *μ*g) at monthly intervals [25]. Vitetta et al. demonstrated that RiVax was safe and elicits neutralizing antibody in a cell based assay. Vitetta et al. reported that in the low-dose group, one out of five had neutralizing antibody, in the intermediate dose group four out of five had neutralizing antibody, and in the high-dose group five out of five had neutralizing antibody. In the two higher dose groups, neutralizing antibody titers were similar but somewhat modest. Vitetta et al. estimated that the vaccine could protect against an injected dose of ricin of (0.3−3.0 mg) or approximately 1 to 10–fold the human LD_50_. However, the duration of antibody titers after three vaccinations (range: 14–127 days) was suboptimal and not related to dosing group.

While initial RiVax phase I results were encouraging, vaccine formulation and stability remain problematic. Vitetta et al. required the use of four different vaccine lots during the course of the initial 15 subject phase I study [25]. Moreover, RiVax formulation required storage at −70°C in a buffer containing 50% glycerol. Therefore, Smallshaw and Vitetta subsequently developed a lyophilized formulation of the vaccine that retained immunogenicity when stored at 4°C [26, 27].

A second RiVax phase I trial in 30 subjects at three different dose levels, utilizing an alum adjuvant formulation, was supported by an FDA Orphan Products grant to University of Texas Southwestern (UTSW). As of March 29, 2011, enrollment for the second phase I trial [28] was complete [29]. In their SEC annual report filing, Soligenix reported that preliminary results from the second phase 1 trial indicated that RiVax appeared safe at all doses tested. To date, human immunogenicity data have not been reported. Soligenix also reported that they initiated a comprehensive program to evaluate the efficiency of RiVax in nonhuman primates at the Tulane University Health Sciences Center [29].

### 4.3. RVEc Recombinant Vaccine

USAMRIID has developed a recombinant RTA vaccine 1–33/44–198 (rRTA 1–33/44–198) (RV*Ec*) produced in *Escherichia coli *[30–32]. Based on preclinical studies, including a pivotal repeated-dose toxicology study in New Zealand white rabbits conducted under GLP [33], this product was determined to have a reasonable safety profile for use in human studies. The pre-clinical testing demonstrated no detectable ribosome inactivating protein (RIP) activity [33] or evidence of vascular leak syndrome (VLS) [34]. A phase I (*N* = 30) first in human escalating, multiple-dose, and single-center study to evaluate the safety and immunogenicity of RVEc was launched at USAMRIID, Fort Detrick MD, in April 2011. The phase I study is expected to be completed by the first half of 2013 [35, 36].

## 5. Monoclonal Antibody Pre-Clinical Development and Proof of Concept for Postexposure Prophylaxis

Neal et al. reported that passive prophylactic administration (intraperitoneal {IP} injection) of GD12 (a murine IgG1 monoclonal antibody (Mab)—anti-RTA) when administered 24 h prior to challenge was sufficient to protect mice against intraperitoneal ricin challenge of 5 LD_50_ [37]. Neal et al. further demonstrated that GD12 protected mice utilizing a backpack tumor delivery system after intragastric ricin challenge of 5 mg/kg. Neal et al. did not test GD12 in the setting of post-exposure prophylaxis. In a follow-up study, Neal et al. demonstrated similar protection in mice when two other monoclonal antibodies, R70 (anti-RTA) and 24B11 (anti-RTB), were passively administered using the so-called backpack tumor model [38]. The mice then survived challenge with intragastric ricin 5 mg/kg 12–24 h. In addition, R70 Mab protected mice after it was administered IP, 12–24 h before intragastric ricin challenge of 5 mg/kg.

Prigent et al. demonstrated that a combination of three Mabs (2 anti-RTB and 1 anti-RTA) to ricin protected mice when the three Mabs were administered intravenously (IV) within 7.5 h after ricin intranasal challenge of 5 LD_50_ [39]. Thus, it would appear that Prigent et al. demonstrated a proof of concept for effective post-exposure prophylaxis to lethal-dose intranasal challenge to ricin [39].

## 6. Small Molecule Inhibitors: Preclinical Development and Pre-Exposure Prophylaxis

Stechmann et al. have recently reported on the successful identification of a selective small molecule inhibitor, Retro-2, that protected mice in a ricin nasal challenge model, when Retro-2 was administered IP one hour prior to challenge [40]. This small molecule inhibitor is attractive in that it does not act on the toxin itself, but rather it blocks retrograde transport of the toxin, a host-toxin interaction. Stechmann et al. argue that since Retro-2 blocks retrograde transport and does not act on the toxin or the host cell itself, there is a decreased likelihood that significant drug resistance will develop to Retro-2. Moreover, Retro-2 appears to be nontoxic to HeLa cells. Small molecules inhibitors offer another promising potential avenue for the development of effective prophylaxis against ricin toxin exposure [41].

## 7. Rationale for Continued Development of Ricin Countermeasures

Schep et al. have recently argued somewhat simplistically that although ricin is toxic, it does not deserve to be a priority in biological countermeasure development [9]. They maintain that bioterrorists do not possess the technical and logistical skills necessary to formulate and mill ricin powder. St. Georgiev similarly maintained that ricin is more compatible with a tool of assassination instead of a weapon of mass destruction [42]. However, Radosavljevic and Belojevic have recently formulated a much more compelling and comprehensive approach to biodefense prioritization and risk assessment [8]. Their approach incorporates all of the potential biothreat agents on the CDC biothreat agent list. Furthermore, their model considers quantitative and qualitative parameters in assessing risk and has four main components: perpetrators (government institutions/organizations, terrorist groups, individuals); agent (CDC categories A, B, and C); means and media of delivery (air, food, water, fomites); target (direct and indirect) [8]. 

The US Armed Forces, Department of Homeland Security (DHS) personnel, first responders, FBI, local law enforcement personnel, CDC/HHS, the Environmental Protection Agency (EPA), and environmental clean-up crews all need adequate protection against potential biological warfare and bioterrorism. Therefore, there is a critical need for continued development of ricin countermeasures.

## 8. Conclusion

While small molecule inhibitors and Mabs for post-exposure treatment are still being evaluated in a pre-clinical setting, RiVax has been studied in two phase I clinical trials, and RVEc is currently in a phase I human trials. A safe and effective prophylactic vaccine against ricin that is FDA approved for “at risk” individuals should be an important first step in countering this 120-year-old threat.
